# What Characteristics Confer Proteins the Ability to Induce Allergic Responses? IgE Epitope Mapping and Comparison of the Structure of Soybean 2S Albumins and Ara h 2

**DOI:** 10.3390/molecules21050622

**Published:** 2016-05-12

**Authors:** Youngshin Han, Jing Lin, Ludmilla Bardina, Galina A. Grishina, Chaeyoon Lee, Won Hee Seo, Hugh A. Sampson

**Affiliations:** 1 Division of Pediatric Allergy and Immunology and the Jaffe Food Allergy Institute, Icahn School of Medicine at Mount Sinai, New York, NY 10029, USA; jing.s.lin@mssm.edu (J.L.); luda.bardina@mssm.edu (L.B.); galina.grishina@mssm.edu (G.A.G.); hugh.sampson@mssm.edu (H.A.S.); 2 Department of Medical Science, Sungkyunkwan University School of Medicine, Seoul 06351, Korea; 3 Department of Food Science and Engineering, Ewha Woman’s University, Seoul 03760, Korea; dntree03@naver.com; 4 Department of Pediatrics, Korea University College of Medicine, Seoul 02841, Korea

**Keywords:** Ara h 2, epitope mapping, peptide microarray, soybean, 2S albumins

## Abstract

Ara h 2, a peanut 2S albumin, is associated with severe allergic reactions, but a homologous protein, soybean 2S albumin, is not recognized as an important allergen. Structural difference between these proteins might explain this clinical discrepancy. Therefore, we mapped sequential epitopes and compared the structure of Ara h 2, Soy Al 1, and Soy Al 3 (Gly m 8) to confirm whether structural differences account for the discrepancy in clinical responses to these two proteins. Commercially synthesized peptides covering the full length of Ara h 2 and two soybean 2S albumins were analyzed by peptide microarray. Sera from 10 patients with peanut and soybean allergies and seven non-atopic controls were examined. The majority of epitopes in Ara h 2 identified by microarray are consistent with those identified previously. Several regions in the 2S albumins are weakly recognized by individual sera from different patients. A comparison of allergenic epitopes on peanut and soybean proteins suggests that loop-helix type secondary structures and some amino acids with a large side chain including lone electron pair, such as arginine, glutamine, and tyrosine, makes the peptides highly recognizable by the immune system. By utilizing the peptide microarray assay, we mapped IgE epitopes of Ara h 2 and two soybean 2S albumins. The use of peptide microarray mapping and analysis of the epitope characteristics may provide critical information to access the allergenicity of food proteins.

## 1. Introduction

The ability of some proteins to induce an allergic reaction in susceptible individuals is well established. However, it is not clear what characteristics confer various proteins the ability to induce allergic responses or why some proteins are more allergenic than others. Many factors have been suggested to contribute to the overall allergenicity of any given protein, such as the number of IgE-binding epitopes, resistance to heat and proteolysis, the glycosylation status and enzymatic activity [1,2]. The presence of certain epitopes is essential [3,4], but the fundamental characteristics that are common to protein epitopes are still not delineated. The FAO/WHO recommended a decision tree to evaluate allergenicity of novel proteins that include digestibility and greater than 35% sequence similarity [5].

2S albumins are known to be intrinsically more allergenic than many other proteins [6,7,8]. They contain the conserved cysteine skeleton CXnCXnCCXnCXCXnCXnC held together by four disulfide bonds, which gives 2S albumins greater stability during proteolytic digestion and heat/chemical treatment. Many 2S albumins from different species have been identified as allergens, such as those in Brazil nuts, sesame seeds, walnuts, rapeseeds, cashew nuts, peanuts, soybean, and mustard [9,10,11,12,13,14,15,16,17].

Peanut allergen Ara h 2, soybean 2S albumin 1 (Soy Al 1, Accession #: AAD09630), and soybean 2S albumin 3 (Soy Al 3, Accession #: AAD00178, allergen name: Gly m 8), which belong to the 2S albumin family, are evolutionarily close to each other. Sequence homology between peanut and soybean 2S albumins is about 40% and all of them are stabile during proteolytic digestion; however, their allergenic behaviors are very different [18,19]. Ara h 2 is associated with severe allergic reactions, but soybean 2S albumin 1 and 3 are less important than Ara h 2. The soybean 2 S albumins differ from other albumins because of their unusually high content of charged amino acids, such as lysine (Soy Al 1: 9.0%, Soy Al 3: 9.5%), aspartic acid (Soy Al 1: 4.5%, Soy Al 3: 9.5%), and glutamic acid (Soy Al 1: 12.3%, Soy Al 3: 10.8%), whereas Ara h 2 contains a high proportion of nitrogen-containing amino acids such as glutamine (14.7%) and arginine (10.9%). Protein–protein binding is a noncovalent interaction mediated by residues found in both proteins. Some residues may contribute to the favorable binding interaction. A comparison of residues in allergenic and antigenic binding sites could provide insight into why some proteins are major allergens and others are not.

In the present study, we mapped sequential epitopes using peptide microarrays and compared the structure of Ara h 2, Soy Al 1, and Soy Al 3 at different levels such as primary, secondary and tertiary structure. Bioinformatics tools were used to investigate associations with the frequent discrepancies in clinical responses to these two proteins.

## 2. Results

### 2.1. Detection of Sequential Epitopes

In order to compare the sequential epitopes identified on peanut Ara h 2 with soybean 2S albumins, sera from peanut and soybean allergic subjects were screened against three sets of 15 overlapping peptides; each set encompasses the entire Ara h 2, Soy Al 1, and Soy Al 3 protein sequences. The microarray used in this study is a modified method documented previously as a reliable tool to identify sequential IgE epitopes on peanut allergens [20,21]. In order to improve the sensitivity of the method, we changed the detection system from an Alexa to a dendrimer amplification system. Dendrimers are complex, branched molecules built from interconnected DNA monomers. Reproducibility of this method shows an intra-assay correlation coefficient of 0.93.

**Figure 1:**
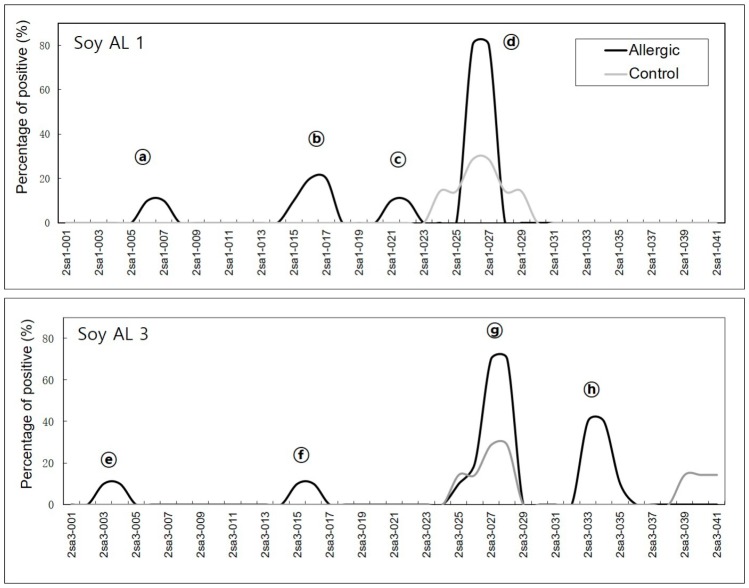
Positive binding in Soy Al 1 and Soy Al 3 are shown as alphabet characters. The *X*-axis of the graph represents each peptide. The *Y*-axis of the graph represents the percentage of positive binding.

**Figure 2:**
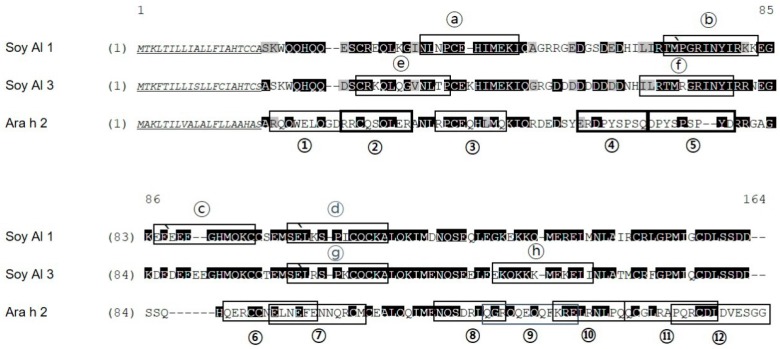
Alignment of Soy Al 1, Soy Al 3, and Ara h 2 amino acid sequences. Identical amino acids have a black background; similar amino acids have a gray background; signal peptides are underlined. The degree of similarity (identity) of Ara h 2 *vs.* Soy Al 1 and Ara h 2 *vs.* Soy Al 3 are 44.2% (35.6%) and 41.5% (34.8%), respectively. Epitopes identified are indicated with a black box. Immunodominant epitopes are indicated with a bold box.

**Figure 3:**
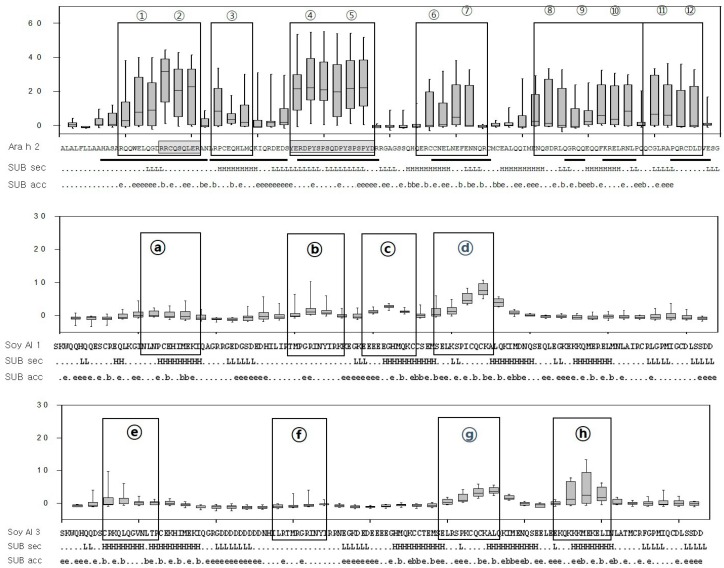
Box-and-whisker plots of the peptides and predicted secondary structure and solvent accessibility. The *X*-axis represents each peptide and the *Y*-axis represents the signal intensity (*Z* score). The median, first, and third quartiles of the *Z* score are shown and vertical lines extend between the 5th and 95th percentile. Sequences of Ara h 2, Soy Al 1, Soy Al 3, and epitopes are shown under each graph. The black boxes represent epitopes identified in the present study and underlines represent those previously defined. Subset of predicted secondary structure (SUB sec) shows the predicted secondary structure of the proteins, where H and L represent helical and loop structures, respectively. Subset of predicted solvent accessibility (SUB acc) shows the likelihood of relative solvent accessibility for proteins, b = 0%–16%, e = 16%–100%.

In soybean 2S albumins, eight regions (a–h) of Soy Al 1 and Soy Al 3 are recognized by patients’ IgE (Figure 1). Epitopes d and g are recognized by normal serum. Therefore, we cannot exclude the possibility of non-specific binding of the two peptides despite significantly high signal intensity in patients. In Ara h 2, twelve sequential IgE binding epitopes are located throughout the length of the amino acid sequence (Figure 2 and Figure 3). They show high positive rate and strong signal intensity. Three of these epitopes, 2, 4, and 5 (RRCQSQLER, ERDPYSPSQ, and DPYSPSPYD), appear to be immunodominant, because peptides containing these epitopes bind the IgE antibody in 90% of the sera tested and have high signal intensity. Overall the pattern of peptide recognition of Soy Al 1 and Soy Al 3 differ markedly from the binding of IgE to equivalent sequential peptides from peanut Ara h 2 (Figure 3). In contrast to Ara h 2, the corresponding sequence of soybean 2S albumins appears to be of minor importance immunologically. The signal intensity and positive rate are much lower than those of Ara h 2.

### 2.2. Structure Comparison

**Figure 4:**
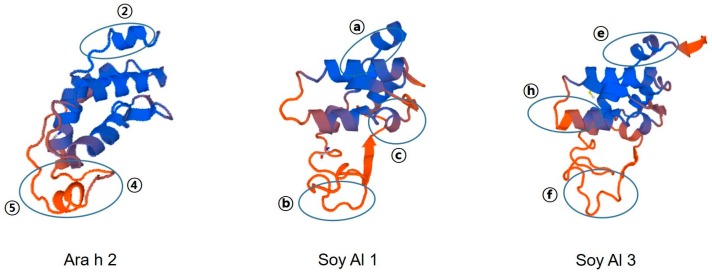
Predicted 3D structure and major epitope of Ara h 2, Soy Al 1, and Soy Al 3. Blue and red colors represent the helix and loop structure, respectively. Identified epitopes are indicated with ovals.

Amino acid sequence-based structure analysis shows that the degree of similarity (identity) between Ara h 2 and Soy Al 1 and Soy Al 3 is 44.2% (35.6%) and 41.5% (34.8%), respectively. The composition of the helix-type secondary structure of Ara h 2, Soy Al 1, and Soy Al 3 are 51.28%, 54.19%, and 56.33%. The compositions of the loop type of Ara h 2, Soy Al 1, and Soy Al 3 are 48.72%, 45.81%, and 43.67%, respectively. Overall, the three proteins have similar primary and secondary values, which suggests that their structures are similar. Three-dimensional images of the three proteins are shown in Figure 4. These three proteins have four helix structures and one large loop structure in common, which makes the overall appearance appear to be similar. However, the position of the helix and folding of the loop differ between Ara h 2 and Soybean albumins (Figure 4). In Ara h 2, three immunodominant epitopes 2, 4, and 5 are located in a protruding loop-helix area (Figure 4) and most epitopes include a loop-helix structure (Figure 3). In Soy Al 1 and Al 3, all epitopes are located in a loop or loop-helix area, which is quite similar to Ara h 2.

**Figure 5:**
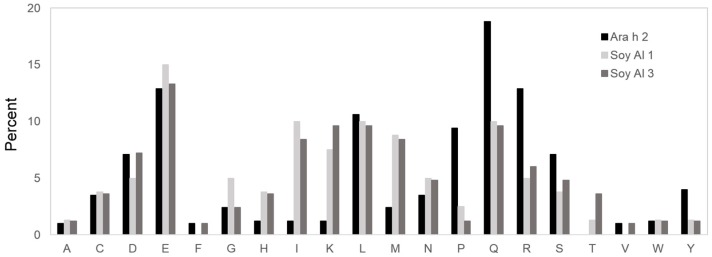
Amino acid comparison of the Ara h 2 epitope and homologous area with Soybean 2S albumins. The *Y*-axis of the graph represents the percentage of each amino acid in epitopes. A = alanine; C = cysteine; D = aspartic acid; E = glutamic acid; F = phenylalanine; G = glycine; H = histidine; I = isoleucine; K = lysine; L = leucine; M = methionine; N = asparagine; P = proline; Q = glutamine; R = arginine; S = serine; T = threonine; V = valine; W = tryptophan; Y = tyrosine.

Based on the comparison of structure, the secondary and tertiary structures are not good enough to explain the difference in reactivity to IgE between Ara h 2 and Soybean albumins. The number of amino acids comprising Ara h 2 epitopes is 105. Amino acids in Ara h 2 epitopes and equivalent peptides of soybean 2S albumins were analyzed for chemical characteristics. The relative contribution of each of the 20 amino acid types to the Ara h 2 epitopes and corresponding peptide in soybean 2S albumins are presented in Figure 5, which shows a difference in amino acid composition among the proteins. Arginine (R), glutamine (Q), proline (P) and tyrosine (Y) appear two times more in Ara h 2 epitopes compared to that in the soybean 2S albumins. Notably, immunodominant epitopes, 2, 4, and 5 (RRCQSQLER, ERDPYSPSQ, and DPYSPSPYD) have a high percentage (56%) of R, Q, P, and Y amino acid; whereas, corresponding areas in Soy Al 1 and Soy Al 3 (see Figure 3) have a low percentage of R, Q, P, and Y amino acids (18% in Soy Al 1 and 22% in Soy Al 3).

## 3. Discussion

The characterization of IgE epitopes of food allergens is fundamental to the understanding of mechanisms responsible for food allergies. In this study, we were interested in determining which structural characteristics of peptides are important for determining IgE binding epitopes. We chose Ara h 2 and soybean 2S albumins due to their apparent difference in clinical allergenicity and similarity in protein stability and sequence. First, we tried to identify the allergenic epitopes of Ara h 2 and soybean 2S albumins utilizing a microarray technology, which employed a sensitive Dendrimer detection system. Then, we analyzed the structural features of the epitopes on the two proteins to determine which structural characteristics might endow the protein with its allergenic properties. Patients clinically reactive to both soybean and peanut concomitantly were used so that IgE binding was comparable. We found eight areas on soybean 2S albumins that are recognized by IgE and some structural features of epitopes on Ara h 2 that differ from Soy Al 1 and Soy Al 3. This analysis suggests that loop-helix type secondary structures and some amino acid such as Arginine, glutamine, proline, and tyrosine on the food protein surface may be important factors in determining the IgE binding sites.

In Ara h 2, twelve sequential IgE binding epitopes, three of which are immunodominant epitopes, are identified. These results coincide with allergenic epitopes reported in previous studies [22,23,24]. In contrast to Ara h 2, only a few peptides of Soy Al 1 and Soy Al 3 are bound by IgE antibody from the sera tested and have very low signal intensity. These results suggest that IgE antibodies reactive to Ara h 2 barely bind to Soy Al 1 and Soy Al 3. Soy Al 1 and Soy Al 3 are predicted to have somewhat similar binding patterns to Ara h 2, because they have greater than 40% sequence similarity with Ara h 2 and are stabile during proteolytic digestion. However, when we closely examined the epitopes, we found inconsistencies in comparable sequences between soybean 2S albumin and Ara h 2. The three immuno-dominant epitopes, 2, 4, and 5 have approximately 20% sequence similarity, while the two immuno-intermediate epitopes 3 and 8 of Ara h 2 have about 60% sequence similarity with Soy Al 1 and Soy Al 3. It is believed that the higher the degree of sequence similarity, the greater likelihood of cross-activity. However, in the results shown here, the immunodominant areas with low degrees of sequence similarity show weak binding to the corresponding area in the soybean 2S albumins. On the other hand, Ara h 2 epitope 3 and 8 have a high degree of sequence similarity in soybean, but they do not bind to soybean 2S albumins. These findings indicate that greater than 40% sequence similarity does not necessarily predict similar IgE binding patterns to allergens or to cross-reactivity of allergens; the data further suggest that there are other important factors in determining allergenicity.

The characteristics that actually endow an antigenic or allergenic property are the center of much debate [8,25,26,27]. Several studies suggest traits that determine epitopes, such as hydrophilicity, accessibility of the surface area, protrusion of sites from the protein surface, structural complementarity in the antibody–antigen interface, amino acid preference, secondary structure preference, geometry, *etc.* [28].

In our studies, most epitopes include the loop-helix structure, which is consistent with previous studies [29,30,31,32]. Annick-Barre, *et al.* show that 10 sequential B-cell epitopes on Ara h 2 are primarily displayed on the molecular surface, especially the three immunodominant epitopes, which are located in a protruding extra loop [33]. Alcocer, *et al.* mapped the epitopes of Brazil nut allergen, Ber e 1, onto a helix-loop-helix region [31]. Parvalbumin, which is as allergen in fish, is characterized by a conservative helix-loop-helix structure [34]. These results suggest that the loop-helix type secondary structure on the surface is an important factor in determining antigen–antibody interactions.

The epitopes of peanut are enriched with polar amino acids. These results are similar to previous reports, which show that polar residues are commonly found on surfaces and within epitopes [28,35]. Arginine, glutamine, proline, and tyrosine appear two times more in Ara h 2 epitopes than the soybean 2S albumins. These amino acids, except proline, have large side chain residues with a lone electron pair. The important role of arginine and tyrosine in antigen–antibody binding is supported by a previous report [36] claiming that one positively charged amino acid, arginine, and two hydrophobic aromatic residues, tryptophan and tyrosine, are generally preferred in protein–protein interactions due to their ability to form multiple interactions. Interestingly, the positive charged lysine is similar to arginine in shape, but it appears much less frequently in epitopes. This suggests that a side-chain charge is also an important factor in determining the antigen–antibody binding. Soy Al 1 and Soy Al 3 have an unusually high lysine content, while lysine content in most 2S albumin allergens is only about 1%. A protein-IgE interaction is determined by a chemical bond, thus chemical properties of amino acids on the protein surface could be an important factor in determining the allergenicity of protein.

A peptide microarray assay allows characterization of large numbers of individual patient samples and examination of thousands of peptides on a single slide [21,37]. Simultaneous immunolabeling of proteins/peptides provides comparable conditions between peptides of two proteins, thus eliminating inter-assay variability. In this study, soybean 2S albumins are shown to have minor IgE binding to several epitopes, which differs from that reported in previous papers that have not implicated 2S albumins as allergens [19,38,39,40]. It is possible that our microarray technology is more sensitive than immunoblot and protein microarray assays that were previously used. It is not clear whether this minor binding has clinical meaning.

This study has some limitations. First, the main focus of the study was on identifying some characteristics of food allergy area; therefore, the study result might not be enough to be generalized to other area. Second, the study lacks in identification of epitopes that are associated clinical reactions because of absence of sensitized patients who tolerate peanuts [41]. For the purpose of identification of epitopes associated with clinical reactions, a new study is being undertaken. Third, carbohydrate and lipid are important factors to determine antigen–antibody interaction and therefore our finding is limited to the area of protein [42]. Furthermore, food processes including roasting is not considered in this study.

## 4. Materials and Methods

### 4.1. Patient Sera

Ten patients, who were allergic to soybean and peanut concomitantly, were included in the study (Table 1). Diagnosis of soybean allergy was based on the results of DBPCFC (double-blind placebo-controlled food challenge) or open challenges. A positive peanut-specific IgE assay was necessary for the diagnosis of peanut allergy. Peanut-specific IgE levels were measured with the Phadia ImmunoCAP-system (Uppsala, Sweden). Levels of peanut-specific IgE greater than 15 kU_A_/L were considered predictive of reactivity [43]. Sera from seven healthy, non-atopic individuals, who have never demonstrated any symptoms following the ingestion of peanut or soybeans, were used as negative controls. After informed consent was received, blood samples were obtained and the sera were frozen at −80 °C until used. This study was approved by the Mount Sinai Institutional Review Board.

### 4.2. Microarray

A library of overlapping peptides, consisting of 15 amino acids with an offset of 3, corresponding to the primary sequence of Ara h 2, Soy Al 1 and Soy Al 3, were commercially synthesized using the PepStar^®^ technique by JPT Peptide Technologies (Berlin, Germany). Peptides were printed in two sets of duplicates on SuperEpoxy glass slides (ArrayIt™, TeleChem International, Inc., Sunnyvale, CA, USA) using the NanoPrint™ Microarrayer 60 (TeleChem International, Inc.). Additional array elements included protein printing buffer (TeleChem International, Inc.) as negative control spots for background normalization.

Immunolabeling was performed, as previously described, but with some modifications [21,29,44]. In brief, the slides were blocked with 400 µL of 1% human serum albumin (HSA, Sigma-Aldrich, St. Louis, MO, USA) in phosphate-buffered saline containing 0.05% Tween 20 (PBS-T) for 60 min at room temperature. This step was followed by incubation with 250 µL of patient serum allergic to peanut and soybean concomitantly diluted 1:5 in PBS-T/HSA for 24 h at 4 °C. The slides were then incubated for 24 h at 4 °C with a cocktail of three biotinylated monoclonal human IgE antibodies: one from Invitrogen, Carlsbad, CA, USA and diluted 1:250, one from BD Biosciences Pharmingen, San Jose, CA, USA and diluted 1:250, and one as a gift from Phadia, Uppsala, Sweden, biotinylated in our laboratory and diluted 1:1000. The slides were then incubated for 3 h at room temperature with anti-biotin-Dendrimer_Oyster 550 (0.6 µg/mL) (Genisphere) in Dendrimer Buffer (Genisphere, Hatfield, PA, USA) with the addition of 0.02 µg/mL of salmon sperm DNA (Invitrogen). Finally, the slides were washed with PBS-T, 15 mM Tris buffer, centrifuge dried, washed with 0.1× PBS, centrifuge dried, washed again with 0.05× PBS, and centrifuge dried.

Immunolabeled slides were scanned using a ScanArray^®^Gx (PerkinElmer, Waltham, MA, USA). Images were saved as a TIF format and the fluorescence signal was digitized with ScanArray Express Microarray Analysis System (PerkinElmer), exported as comma-delimited (CSV) files and transformed to z score as previously described [21,43,45]. Based on previous studies [46], an epitope was defined as involving at least 2 overlapping peptides. A single positive peptide was not considered an epitope. Overlapping amino acids were considered epitopes.

### 4.3. Structure Comparison

A comparison of amino acid similarity was performed using Vector NTI Advance 10 software program (Invitrogen). PredictProtein Server was used to predict the consensus secondary structure and the relative solvent accessibility [47]. SWISS-MODEL was used to predict 3D structure [48,49].

## 5. Conclusions

In this study, we have mapped epitopes of Ara h 2, Soy Al 1, and Soy Al 3 using a peptide microarray system, which made it possible to compare the characteristics of various epitopes. Loop-helix type structures and some amino acids with a large side chain including electron lone pair, such as arginine, glutamine and tyrosine, on the surface of the tertiary structure appear to comprise peptides with a higher potential for immune recognition; this seems to play an important role in antigen–antibody interactions. The overlapping peptide microarray system will greatly facilitate screening large numbers of individuals for epitope recognition. In turn, analysis of the characteristics of epitopes will give critical information to assess the allergenicity of various proteins. Our finding in this study might be limited in the area of food allergy and suggested one factor to determine antigen–antibody interaction. Therefore, further studies to identify the characteristics of epitopes and the factors to determine of antigen–antibody interaction are needed.

## Figures and Tables

**Table 1:** Characteristics of peanut and soybean-allergic subjects. AD: atopic dermatitis, AR: allergic rhinitis.

No.	Age (year)	Challenge Type	Soybean Specific IgE (kU_A_/L)	Peanut Specific IgE (kU_A_/L)	Other Atopy
1	4	Open	12.7	100	None
2	4	Single Blind	5.31	33.3	None
3	4	Open	77.7	100	AD, asthma
4	4	Double Blind	16.1	57.3	AD, asthma
5	4	Double Blind	77.4	100	AD, asthma
6	5	Open	0.53	17.1	AD, asthma
7	6	Double Blind	16.9	100	AD, asthma
8	7	Double Blind	35.3	100	AD, AR, asthma
9	9	Double Blind	6.64	15.4	AR, asthma
10	9	Double Blind	19.6	100	AD, AR, asthma

## Abbreviations

Soy Al 1	soybean 2S albumin 1
Soy Al 3	soybean 2S albumin 3
